# Multi-session transcutaneous auricular vagus nerve stimulation for Parkinson's disease: evaluating feasibility, safety, and preliminary efficacy

**DOI:** 10.3389/fneur.2023.1210103

**Published:** 2023-07-18

**Authors:** Daniel H. Lench, Travis H. Turner, Colin McLeod, Heather A. Boger, Lilia Lovera, Lisa Heidelberg, Jordan Elm, Anh Phan, Bashar W. Badran, Vanessa K. Hinson

**Affiliations:** ^1^Department of Neurology, Medical University of South Carolina, Charleston, SC, United States; ^2^Department of Neurology, Augusta University Medical Center, Augusta, GA, United States; ^3^Department of Neurosciences, Medical University of South Carolina, Charleston, SC, United States; ^4^Department of Public Health Sciences, Medical University of South Carolina, Charleston, SC, United States; ^5^Department of Psychiatry, Medical University of South Carolina, Charleston, SC, United States

**Keywords:** transcutaneous auricular vagus nerve stimulation, Parkinson's disease, vagus nerve, taVNS, non-invasive brain stimulation

## Abstract

**Background:**

In pre-clinical animal models of Parkinson's disease (PD), vagus nerve stimulation (VNS) can rescue motor deficits and protect susceptible neuronal populations. Transcutaneous auricular vagus nerve stimulation (taVNS) has emerged as a non-invasive alternative to traditional invasive cervical VNS. This is the first report summarizing the safety, feasibility, and preliminary efficacy of repeated sessions of taVNS in participants with PD.

**Objectives:**

To evaluate the feasibility, safety, and possible efficacy of taVNS for motor and non-motor symptoms in mild to moderate PD.

**Methods:**

This is a double-blind, sham controlled RCT (NCT04157621) of taVNS in 30 subjects with mild to moderate PD without cognitive impairment. Participants received 10, 1-h taVNS sessions (25 Hz, 200% of sensory threshold, 500 μs pulse width, 60 s on and 30 s off) over a 2-week period. Primary outcome measures were feasibility and safety of the intervention; secondary outcomes included the MDS-UPDRS, cognitive function and self-reported symptom improvement.

**Results:**

taVNS treatment was feasible, however, daily in-office visits were reported as being burdensome for participants. While five participants in the taVNS group and three in the sham group self-reported one or more minor adverse events, no major adverse events occurred. There were no group differences on blood pressure and heart rate throughout the intervention. There were no group differences in MDS-UPDRS scores or self-reported measures. Although global cognitive scores remained stable across groups, there was a reduction in verbal fluency within the taVNS group.

**Conclusions:**

taVNS was safe, and well-tolerated in PD participants. Future studies of taVNS for PD should explore at-home stimulation devices and optimize stimulation parameters to reduce variability and maximize engagement of neural targets.

## 1. Introduction

Parkinson's disease is the second most common neurodegenerative disorder and is characterized by a loss of nigrostriatal dopamine cells which becomes more widespread across neural networks with disease progression (1). PD is highly heterogeneous in its presentation, but characteristically involves motor symptoms including resting tremor, bradykinesia, rigidity, and postural instability. While clinical diagnosis is based on clinical symptoms, definitive diagnosis can only be performed post-mortem and requires the identification of Lewy bodies produced by protein aggregates including alpha-synuclein (2). Traditional pharmacological treatments for PD such as levodopa/carbidopa primarily target the underlying depletion of nigrostriatal dopamine (3). Limitations of dopaminergic therapy include the development of motor fluctuations, poor efficacy on a subset of non-motor PD symptoms, and no influence on the rate of disease progression (2, 4, 5). Motor fluctuations in PD can be addressed through a neurosurgical neuromodulation technique known as Deep Brain Stimulation (DBS). DBS uses continuous high frequency stimulation to target either the subthalamic nucleus (STN) or globus pallidus internus (GPi). Despite the effectiveness of this approach, it may not be appropriate for all patients and the responsiveness to DBS is strongly dependent upon responsiveness to dopaminergic medications (4). Furthermore, medications and DBS produce limited improvements in non-motor PD symptoms which have significant impact on patient quality of life (5). In addition to neurodegeneration of dopaminergic substantia nigra (SN) neurons, PD results in significant neuronal loss within the noradrenergic Locus Coeruleus (LC) and cholinergic basal forebrain (6). Neuronal degeneration within these regions appear to precede the onset of PD motor symptoms (7). Although adrenergic and cholinergic projections influence motor control (8, 9), they additionally have projections to limbic and cortical regions which, if disrupted, can result in symptoms such as apathy, fatigue, REM behavior disorder and cognitive decline (10, 11), necessitating development of non-dopaminergic approaches to the management of PD.

Several neuromodulation modalities beyond DBS including transcranial magnetic stimulation (TMS) and transcranial direct current stimulation (tDCS) have been investigated as adjuvant therapies to address PD symptoms, however, these approaches are limited in their ability to target deep brain structures affected early in PD pathology (12). The vagus nerve is the longest cranial nerve in the body and carries both sensory afferent information from internal organs to the brain and efferent motor signals from the brain to the body (13). Uniquely, vagus nerve stimulation (VNS) can modulate cholinergic and noradrenergic outputs indirectly via the nucleus tractus solitarius (NTS) (14, 15). Furthermore, preclinical animal models of PD have demonstrated VNS can improve locomotor control, reduce markers of neuroinflammation, decrease intrasomal alpha synuclein, increase brain derived neurotrophic factor (BDNF) and attenuate neuronal damage within the LC and SN (16, 17). Thus, VNS may be an effective approach to improve motor and non-motor symptoms in people with PD. Although cervical VNS is relatively safe, there are risks associated with surgical implantation, and costs of the procedure can be high, reducing patient access (18). Non-invasive forms of brain stimulation can target these structures and minimize the risks associated with electrode implantation. Transcutaneous auricular VNS (taVNS) is a non-invasive form of VNS which delivers electrical stimulation to the auricular branch of the vagus nerve (ABVN) (19, 20). Previous taVNS studies across a range of sites and protocols have reported stimulation to be safe, with limited side effect profiles which most frequently includes transient ear pain, headache, and tingling, however, evidence of safety and efficacy in the PD population is limited (21). Early reports have suggested taVNS treatment may be effective in treating gait disability in PD, however, these studies have been limited to a single session of stimulation (22). The only non-invasive VNS study in PD performed over multiple sessions used transcutaneous cervical VNS in a small sample with limited reporting on specific stimulation parameters and outcomes (Table 1). taVNS can activate cerebral afferents of the vagal pathway and modulate physiological markers (i.e., heart rate) as is observed with traditional, cervically implanted VNS (26–28). These taVNS studies provide preliminary evidence for motor and non-motor benefits but may have been limited by low dosing and single session therapy. The objective of this study was to establish the feasibility, safety, and signals of efficacy of taVNS in mild to moderate PD participants using a comprehensive multiday clinical trial.

**Table 1:** Summary of published studies using non-invasive VNS in Parkinson's disease.

**References**	**nVNS type**	**Design**	**Sample**	**Length of stimulation**	**Stim parameters**	**Primary endpoints/ reported findings**
Marano et al. (23)	taVNS	Randomized, double-blind, sham controlled, and crossover	12 taVNS	Single 30-min session	30 s trains, 600 pulses/train (20 Hz) repeated every 4.5 min	Improvements in UPDRS-III and spatiotemporal gait measures
Mondal et al. (24)	Handheld nVNS	Observational, open label, and no sham	19 nVNS	Two 120 s sessions	Not reported	Improvement in spatiotemporal gait measures
Morris et al. (22)	Handheld nVNS	Randomized and sham controlled	15 nVNS, 15 sham	Single session for 120 s	Not reported	Improvement in spatiotemporal gait measures
Kaut et al. (25)	tcVNS	Randomized, double-blind, and sham controlled	10 nVNS, 9 sham	4 weeks of stimulation, 4 sessions/day	Not reported	Improvement in gastrointestinal symptom rating scale

## 2. Materials and methods

### 2.1. Study design

**Figure 1:**
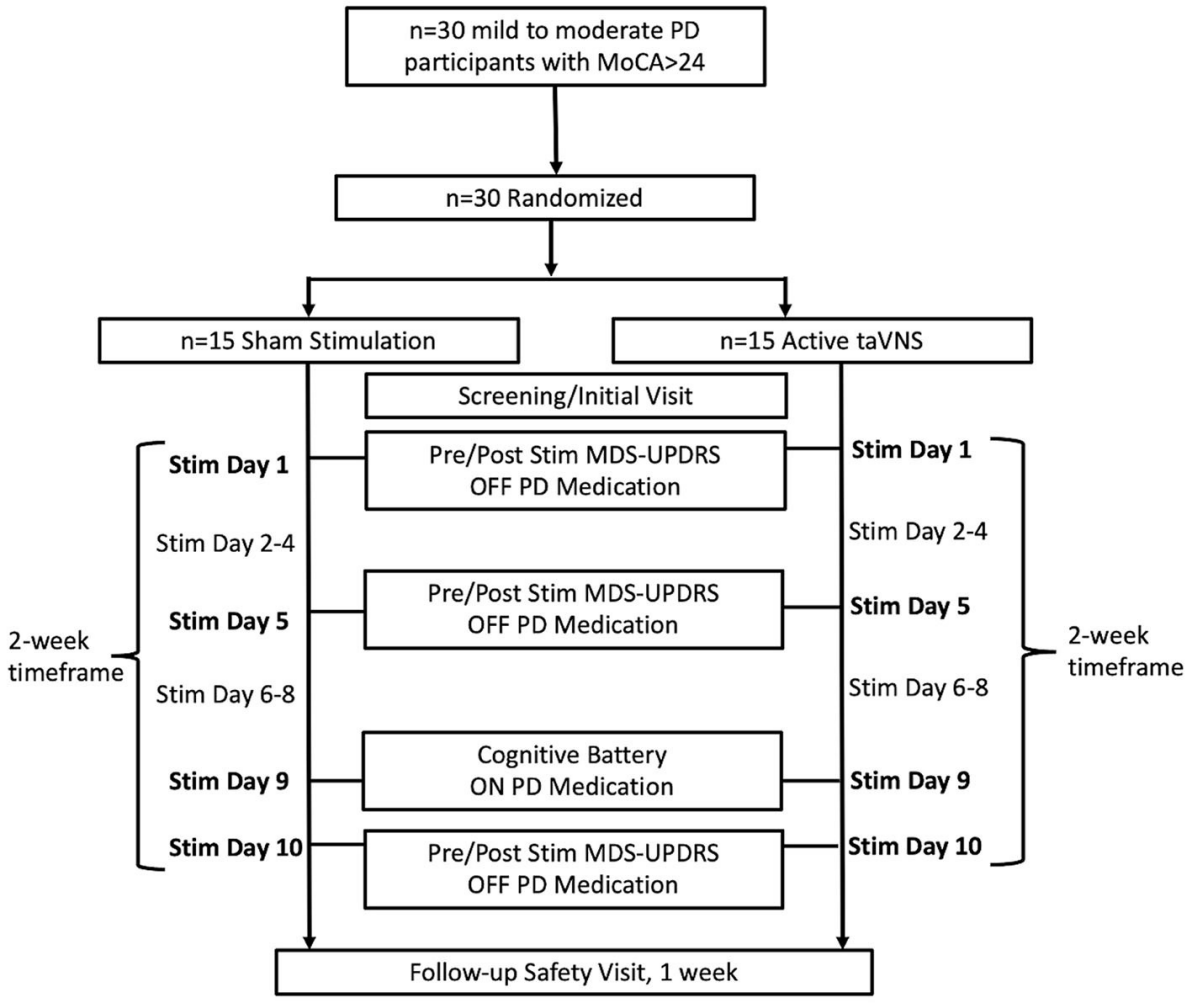
Flow diagram of participant enrollment, treatment group allocation, and timeline of study procedures and assessments.

From 2018 to 2021, 30 participants with mild to moderate idiopathic PD were recruited from a Movement Disorders Clinic at the Medical University of South Carolina (MUSC) to undergo either taVNS (*n* = 15) or sham (*n* = 15) stimulation (Figure 1). The length of participant recruitment was longer than anticipated due to the impact of COVID-19 on in-person visits. Study procedures were reviewed and approved by the MUSC Institutional Review Board and the study was registered on ClinicalTrials.gov (NCT04157621). All participants enrolled in the study were informed of study procedures and provided written consent. Study procedures included an initial screening visit, followed by 10 visits for stimulation over a period of 2 weeks (Figure 1). One week following the final stimulation visit, a follow-up safety assessment was performed. Assessments of motor efficacy were performed in the OFF-medication state in order to avoid any influence of medication response fluctuations on the MDS-UPDRS Part III. Cognitive measures on the contrary were elicited in the ON medication state in order to avoid lack of effort, bradyphrenia, anxiety, and depression, which can be associated with OFF periods, interfering with cognitive assessments.

### 2.2. Randomization and blinding

Randomization of the 30 participants was performed using the REDCap randomization module in which there was a 1:1 ratio in group assignment (taVNS vs. sham stimulation). All participants and study staff were blinded to which treatment assignment with exception of the Principal Investigator and laboratory personnel performing active or sham stimulation. To maintain objectivity of motor assessments a blinded movement disorders neurologist rated videotaped MDS-UPDRS III examinations.

### 2.3. Sample size and power calculations

There are no prior studies from which to estimate treatment or placebo effects associated with taVNS in PD. This study was powered to detect a clinically meaningful reduction of 3.25 points on the MDS-UPDRS Part III (29), assuming a small and statistically non-significant change of 1 on the MDS-UPDRS Part III with sham taVNS, and a pooled standard deviation of 2.0. At 80% power and alpha = 0.05, using a two-tailed *t*-test for difference in means, the total sample size required was 26 (13 per group). Additional participants were recruited to account for attrition.

### 2.4. Inclusion and exclusion criteria

Individuals meeting UK Brain Bank diagnostic criteria for Parkinson's disease (30) who were between 40 and 79 years of age, taking levodopa three or more times daily, with Hoehn and Yahr staging between 2 and 3 were eligible to participate. Exclusion criteria included prior diagnosis of dementia (31) or mild cognitive impairment on screening [MOCA < 24, (32, 33)], visual hallucinations or other psychotic symptoms, history of ear trauma or facial pain disorder, history of comorbid neurologic disorders or major cardiovascular conditions, history of deep brain stimulation or other brain surgery, neurogenic orthostatic hypotension, chronic respiratory illness, pregnancy, and use of cholinesterase inhibitors or Level 2 and 3 anticholinergic medications (34). All participants were expected to be stable on medication for PD motor and non-motor symptoms for a minimum of 30 days before and for the duration of the trial.

### 2.5. Transcutaneous auricular vagus nerve stimulation (taVNS) protocol

**Figure 2:**
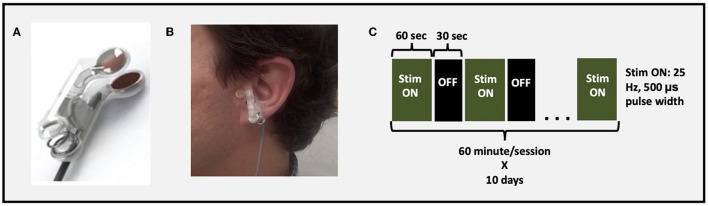
taVNS setup and parameters. **(A)** taVNS electrodes used to deliver stimulation, **(B)** example of taVNS electrode device attached to tragus, **(C)** stimulation parameters and paradigm.

Active taVNS was applied using custom fabricated ear clip electrodes (1 cm round electrode surface) designed to deliver electrical stimulation to the anterior wall of the left outer ear canal landmarked by the tragus (Figure 2). The reasoning for left ear stimulation is based on conventional, surgically implanted VNS trials which primarily target the left cervical bundle of the vagus nerve. In addition, the afferent effects of taVNS have been primarily established in a left-only fashion (35–37). Sham stimulation utilized the same stimulation parameters; however current was delivered to the left earlobe, a region with limited to no innervation of the ABVN. Electrodes were applied to the ear using a conductive Ten20 paste and connected to an FDA 510 k-cleared constant current electrical nerve stimulation device.

After participants were connected to the stimulation system, they remained supine and were instructed to stay awake and maintain still in a comfortable position. Stimulation was administered for 1-h/day for 10 total days spread over 2 weeks. The stimulation parameters were consistent with prior work suggesting activation of vagal afferent network (36): Pulse Width 500 μs; frequency 25 Hz; duty cycle 60 s On, 30 s OFF; current intensity 200% perceptual threshold (Figure 2). Perceptual threshold was defined by the minimum amount of current required to be perceived by the participant.

### 2.6. Safety and tolerability evaluation

Safety and tolerability of taVNS stimulation included monitoring of participant reported adverse events, MDS UPDRS Part III examinations, cognitive testing, and the Columbia-Suicide Severity Rating Scale (C-SSRS). Monitoring of vital signs (heart rate and blood pressure) occurred pre-stimulation, 10 min into stimulation and 50 min into stimulation. Neurogenic orthostatic hypotension (NOH) was reported as an adverse event if it was clinically significant. Clinically significant NOH was defined as lightheadedness or syncope in the setting of a 20 mm Hg or more drop in systolic BP or 10 mm Hg or more drop in diastolic BP with standing at 50% or more of assessments. An adverse event was considered related to the study intervention if the event had a reasonable possibility of being causally related to the intervention being administered. Qualitative interviews following study participation were performed to assess participant experiences during the study and feasibility of developing taVNS as a clinical intervention.

### 2.7. Secondary outcomes

As an early indicator for treatment efficacy, we assessed motor function with the Movement Disorder Society Unified Parkinson's Disease Rating Scale (MDS-UPDRS) Part III, a comprehensive scale that rates the severity of motor symptoms in Parkinson's Disease, modified for videotaped assessments (rigidity testing omitted) (38). Both acute (pre to post stimulation change on a given visit) and subacute (baseline to visit 5 and visit 10 change) assessments were evaluated. All MDS-UPDRS Part III evaluations were performed in the OFF-medication state (defined as at least 12 h without PD medication) to capture the standalone benefits of stimulation independent of dopaminergic medication. MDS-UPDRS Parts I, II, and IV were used to evaluate changes in motor and non-motor aspects of daily living and motor complications. Other secondary outcome measures focused on changes in cognitive function as measured by the Delis-Kaplan Executive Function System (DKEFS) to evaluate letter fluency, category fluency and category switching (39) and the Digit Span (Backward and Forward) assessment to evaluate short-term and working memory (40). Cognitive secondary outcome measures were obtained in the medication ON-state at baseline and visit 9. Patient reported outcomes included the Movement Disorders Society Non-Motor Symptoms Scale for Parkinson's Disease (NMSS), the freezing of gait questionnaire (FOG-Q) (41, 42), and the Conners Adult ADHD Rating Scale short form self-report (CAARS-S:S). The CAARS-S:S served as a patient reported outcome of symptoms associated with inattention and executive dysfunction (43, 44). Additionally, the Patient-Reported Outcomes Measurement Information System (PROMIS) was used to evaluate sleep related impairment (PROMIS-Sleep Related Impairment), applied cognitive abilities (PROMIS-Applied Cognition) and fatigue (PROMIS-Fatigue) (45). Self-reported questionnaires and outcome measures were collected at screening and visit 9.

### 2.8. Statistical analysis

Demographic data, disease severity measures, motor scores at baseline and non-motor scores at baseline were compared between the two treatment groups (active vs. sham taVNS) using chi-squared and two-sided two-sample *t*-tests. Treatment group differences were considered significant if *p*-value < 0.05. To assess the effects of taVNS on systolic blood pressure, diastolic blood pressure and heart rate, the change in score before and after 50 min of treatment was compared between the taVNS and sham group using a two-sample *t*-tests. Data was aggregated from 112 sham sessions and 122 taVNS sessions where complete vital datasets were available. To assess the effects of taVNS on motor (MDS-UPDRS Part III scores) and cognitive outcome measures, the change in score was compared between the active and sham groups using a two sample-sample *t*-test. Acute effects of taVNS on MDS-UPDRS Part-III scores were evaluated as post-stimulation scores minus pre-stimulation scores at a given visit. Meanwhile subacute effects of taVNS on MDS-UPDRS Part-III scores were evaluated as post-stimulation scores at visit 10 minus pre-stimulation scores at visit 1. The motor outcome measure was considered significant if *p*-value < 0.05. Cognitive outcome measures were considered significant if *p*-value < 0.01 (Bonferroni corrected for the five cognitive scores evaluated). To address missing data statistical analyses were performed by excluding participants with missing data and were then repeated using imputation of missing data points.

## 3. Results

### 3.1. Demographics and baseline data

Table 2 displays the demographics, disease severity and baseline motor and cognitive scores of participants in the taVNS (*n* = 15) and sham (*n* = 15) treatment groups. Fifty percent of participants were female and the mean age was 67. Mean time since symptom onset was 7.7 years (median: 6.5) and mean time since PD diagnosis was 5.1 years (median: 4). Treatment groups did not significantly differ in any demographic characteristics, baseline motor symptom severity (MDS-UPDRS Part-III score active group 25.9 ± 10.3 and sham 24.5 ± 7.3), or baseline cognitive function. Stimulation intensity determined by each participant's sensory threshold was greater in the taVNS group (*t* = 3.32, df = 28, *p*-value = 0.0025) than the sham group. Sensory thresholds ranged from 1.4 to 3 in the taVNS group and 0.8–2 in the sham group (Supplementary Figure 1).

**Table 2:** Demographics and baseline measures.

**Demographic and baseline variables**	**taVNS**	**Sham**
	***n* = 15**	***n* = 15**
Age in years, mean (SD)	65.4 (7.6)	68.4 (7.9)
Male sex, count (%)	7 (46.7%)	8 (53.3%)
White race, count (%)	15 (100.0%)	15 (100.0%)
Not Hispanic or Latino, count (%)	15 (100.0%)	15 (100.0%)
Years of education, mean (SD)	16.9 (2.2)	16.7 (2.0)
Years since PD diagnosis, median (IQR)^a^	4.3 (4.0)	4 (4))
Years since symptom onset, median (IQR)^b^	7 (8)	6 (5)
H and Y score, median (IQR)	2 (0)	2 (0)
MOCA total score, median (IQR)^a^	29.0 (2.0)	28.0 (4.0)
MDS-UPDRS part I sub-score, mean (SD)^a^	8.2 (5.0)	7.4 (4.9)
MDS-UPDRS part II sub-score, mean (SD)^a^	6.6 (4.7)	6.8 (5.7)
MDS-UPDRS part III sub-score, mean (SD)^a^	25.9 (10.3)	24.5 (7.3)
MDS-UPDRS part IV sub-score, mean (SD)^b^	3.0 (2.5)	2.7 (3.2)
DKEFS letter fluency, mean (SD)^d^	50.3 (11.1)	48.7 (13.7)
DFEFS category fluency, mean (SD)^d^	42.8 (8.1)	40.5 (9.4)
DKEFS category switching, mean (SD)^d^	14.4 (3.1)	14.4 (2.7)
Digit span forward total score, mean (SD)^d^	12.0 (1.8)	11.4 (2.5)
Digit span backward total score, mean (SD)^d^	9.2 (2.6)	9.0 (2.9)
CAARS, mean (SD)	18.0 (5.8)	19.5 (8.6)
Non-motor symptom total score, mean (SD)^c^	29.5 (19.1)	34.4 (33.9)
FOG-Q, mean (SD)	3.9 (5.3)	2.4 (4.8)
Fatigue [*T*-score], mean (SD)	49.8 (7.0)	51.0 (9.0)
Sleep related impairment [*T*-score], mean (SD)	49.4 (6.6)	51.3 (8.5)
Applied cognition [*T*-score], mean (SD)	46.8 (9.4)	47.3 (9.9)
Stimulation threshold, mean (SD)^*^	2.0 (0.5)	1.5 (0.3)

Symbols following variable names denote missing data points.

a Data missing from one participant in taVNS group.

b Data missing from two participants in taVNS group.

c Data missing from one participant in sham group.

d Data missing from one participant in taVNS group and one participant in sham group.

* Represents a statistically significant group difference (*p* < 0.05).

### 3.2. Feasibility, adverse events and tolerability of taVNS

All 30 participants in the taVNS and sham treatment groups completed the on-site stimulation visits. The frequency of visits to the study center for stimulation was reported as burdensome by participants via qualitative interviews following trial participation. taVNS administration was well-tolerated and feasible without technical issues at the study site. As far as safety, during the 10-day period of stimulation, five (33.3%) participants in the active taVNS group and 3 (20%) participants in the sham group self-reported one or more minor adverse event (AE). The most frequently reported AE in the active taVNS group was difficulty sleeping (*n* = 2) followed by lightheadedness (*n* = 1), fatigue (*n* = 1), nausea (*n* = 1), ringing in the ear (*n* = 1), grinding teeth (*n* = 1), fluid in the ear (*n* = 1), jitteriness/anxiousness (*n* = 1), and vertigo (*n* = 1). The most frequently reported AE in the sham group was lightheadedness (*n* = 2) followed by difficulty sleeping (*n* = 1), headache (*n* = 1), fatigue (*n* = 1), difficulty concentrating (*n* = 1), and neck pain (*n* = 1). No pain at the site of stimulation was reported for either treatment group. No serious adverse events (SAEs) were reported for the duration of the study and follow-up. At the 1-week follow-up, 2 participants from the active taVNS group reported AEs including stomach and hip pain, difficulty sleeping, fatigue, and constipation. At follow-up, 1 participant from the sham group described decreased hearing within the left ear. There was no evidence for the development of suicidal thoughts as monitored by the Columbia-Suicide Severity Rating Scale (C-SSRS) (46). Vital signs were monitored and remained stable throughout the duration of the study. Systolic blood pressure (t=0.495, df=232, p=0.621), diastolic blood pressure (t=1.374, df=232, p=0.171) and heart rate (t=0.183, df=232, p=0.138) changes from pre-treatment to 50 minutes into treatment did not differ between the sham and taVNS groups. In the taVNS group there was a 3.6 mm Hg drop in systolic blood pressure and a 0.9 mm Hg drop in diastolic blood pressure and a 3.4 bpm drop in heart rate. Meanwhile in the sham group there was a 2.7 mm Hg drop in systolic blood pressure, a 0.7 mm Hg increase in diastolic blood pressure and a 1.8 bpm drop in heart rate. Orthostatic hypotension was assessed throughout the trial was identified to be clinically significant in 1 (6.6%) participant receiving active taVNS and 1 (6.6%) participant receiving sham stimulation.

### 3.3. Motor effects of taVNS

**Figure 3:**
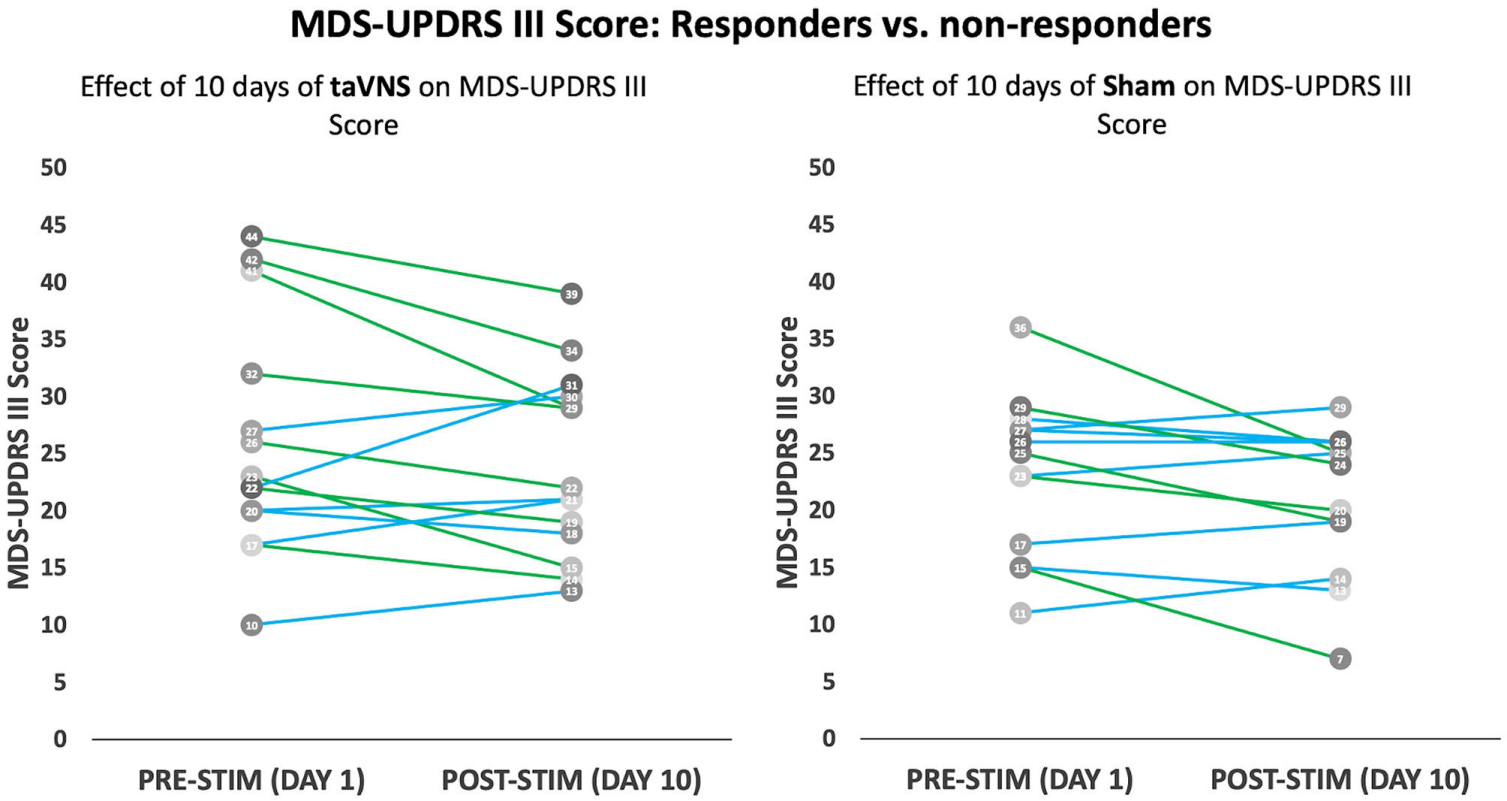
Modified MDS-UPDRS Part III score: MDS-UPDRS Part III scores before and after 10 days of taVNS **(left)** or sham **(right)** is shown for individual participants. Participants with a 3-point or greater improvement in UPDRS Part III are shown as green lines, while those with a <3-point drop are shown in blue lines.

Figure 3 displays individual participant responses with regard to motor symptoms. A detailed summary of results is displayed in Table 3. Participants with missing MDS-UPDRS Part III data at visits 1, 5, and 10 were excluded from the analysis (see Table 3 for sample sizes used in the analysis). Analyses were repeated using imputation of missing data points; however, this is not shown since it did not influence significance of the reported results. Acute effects of stimulation did not significantly differ between groups at any of the timepoints (visits 1, 5, and 10) assessed (*p*-value > 0.05). Both treatment groups showed small reductions in their MDS-UPDRS Part-III scores (2.0 ± 5.6 points reduction in the taVNS group and 2.2 ± 4.3 points reduction in the sham group); however, there were no significant treatment group differences in subacute score changes (*p*-value = 0.906).

**Table 3:** Secondary outcome measures: motor, cognitive, and participant reported outcomes.

	**taVNS**		**Sham**			
**Variable**	***N***	**Mean (SD)**	***N***	**Mean (SD)**	**Mean difference (95% CI)**	***p*** **-value**
**MDS-UPDRS Part III (OFF-state) acute effects**						
Visit 1 (post to pre stim)	13	−0.7 (5.9)	15	−1.6 (4.6)	0.91 (−3.17, 4.98)	0.651
Visit 5 (post to pre stim)	15	−0.5 (3.2)	13	−0.8 (4.1)	0.24 (−2.6, 3.07)	0.865
Visit 10 (post to pre stim)	14	0.9 (3.2)	13	0.0 (4.0)	0.93 (−1.97, 3.82)	0.515
**MDS-UPDRS Part III (OFF-state) subacute effects**						
Visit 10 post to visit 1 pre-stim	14	−2.0 (5.6)	13	−2.2 (4.3)	0.23 (−3.73, 4.19)	0.906
**Other MDS-UPDRS Part I, II, IV subacute effects**						
MDS-UPDRS I (visit 10 to visit 1)	14	0.5 (4.7)	12	0.3 (3.7)	0.17 (−3.29, 3.62)	0.921
MDS-UPDRS II (visit 10 to visit 1)	14	−0.5 (3.1)	12	−0.2 (1.5)	−0.33 (−2.31, 1.64)	0.728
MDS-UPDRS IV (visit 10 to visit 1)	13	−1.1 (2.2)	12	−0.4 (1.6)	−0.66 (−2.24, 0.92)	0.397
**Cognitive battery (ON-state)**						
DKEFS letter fluency	14	−3.8 (7.0)	14	5.4 (9.5)	−9.14 (−15.62, −2.66)	**0.008**
DFEFS category fluency	14	−3.1 (6.9)	14	3.4 (5.0)	−6.5 (−11.16, −1.84)	**0.008**
DKEFS category switching	14	1.3 (2.2)	14	−0.3 (3.1)	1.57 (−0.55, 3.69)	0.140
Digit span forward total score	14	0.2 (1.1)	14	0.7 (1.6)	−0.5 (−1.59, 0.59)	0.355
Digit span backward total score	14	0.1 (1.3)	14	0.5 (1.2)	−0.43 (−1.4, 0.54)	0.372
**Participant reported outcomes measures**						
CAARS (visit 9 to screening visit)	14	1.7 (6.0)	15	0.7 (3.9)	0.98 (−2.84, 4.81)	0.603
NMSS (visit 9 to screening visit)	14	−0.6 (15.2)	12	−5.9 (15.4)	5.27 (−7.16, 17.71)	0.390
FOG-Q (visit 9 to screening visit)	14	0.9 (2.2)	15	0.2 (0.9)	0.66 (−0.67, 1.99)	0.312
PROMIS fatigue [*T*-score] (visit 9 to screening visit)	15	2.6 (6.3)	15	−1.9 (6.9)	4.47 (−0.49, 9.42)	0.076
PROMIS sleep related impairment [*T*-Score] (visit 9 to screening visit)	15	0.7 (6.6)	15	−3.7 (7.7)	4.43 (−0.95, 9.82)	0.103
PROMIS applied cognition [*T*-Score] (visit 9 to screening visit)	15	1.7 (2.9)	15	−0.2 (6.8)	1.93 (−2.06, 5.91)	0.324

Bold values represent significant p-values.

### 3.4. Cognitive effects of taVNS

Cognitive, and patient reported outcomes are presented in Table 3. *Cognition*: As described for the motor outcome, participants with missing DKEFS (letter fluency, category fluency and category switching) data, and missing Digit Span (Backward and Forward) data were excluded from the analysis (taVNS group *n* = 1, sham group *n* = 1). Effects of taVNS on cognitive performance were evaluated by determining treatment group differences in change scores (post-stimulation at visit 9 minus pre-stimulation at baseline). DKEFS letter fluency performance decreased in the taVNS group by 3.8 points but increased in the sham group by 5.4 points. Similarly, DKEFS category fluency performance decreased in the taVNS group by 3.1 points but increased in the sham group by 3.4 points. Change scores in letter fluency (*p*-value = 0.008, uncorrected) and category fluency (*p*-value = 0.008, uncorrected) was significantly reduced in the taVNS group relative to the sham group. After Bonferroni correction accounting for number of cognitive tests performed (*p*-value threshold of 0.01), however, only group differences in category fluency remained significant. DKEFS category switching performance improved in the taVNS group by 1.3 points and was reduced in the sham group by 0.3 points. The Digit Span forward and backward scores increased slightly in the taVNS group (0.2 and 0.1 points, respectively) and increased slightly in the sham group (0.7 and 0.5 point, respectively). There were no significant treatment group differences in change scores for DKEFS category switching, or the digit span forward and backward. *Patient reported outcomes*: No significant changes from screening to visit 9 were observed for the NMSS, the freezing of gait questionnaire, CAARS or the PROMIS questionnaires.

## 4. Discussion

To our knowledge, this is the first prospective, randomized, double-blind, sham-controlled study to assess multi-day taVNS in PD participants. In this study of 30 participants with idiopathic PD, 10 days of taVNS stimulation was feasible, safe and well-tolerated. All participants within the taVNS group completed the full course of stimulation and no major AEs were observed. A minority of participants reported minor AEs, however, these were similar in nature and frequency between taVNS (33.3%) and sham (20.0%) treatment groups. No significant improvements or worsening of overall motor symptoms as measured by MDS-UPDRS Part-III score were observed in the taVNS group. In a subset of responders (>3 point improvement in MDS-UPDRS Part-III) within the taVNS group, bradykinesia and tremor symptoms showed the greatest improvements. Measures of cognition were not found to decline or improve in either group apart from verbal fluency measures which declined to a greater extent among the participants that received taVNS.

In the current study we demonstrated the safety of taVNS in individuals with PD as there were no group differences in the effects of taVNS and sham stimulation on heart rate and blood pressure. This safety profile is particularly important for individuals with PD due to the susceptibility of this population to cardiovascular autonomic dysfunction which can occur with mild to moderate disease progression (47). Furthermore, there were no significant acute or subacute changes in measures of motor function between the taVNS and sham stimulation groups; however, within the taVNS group several participants demonstrated clinically meaningful improvements. These findings indicate that there is a substantial degree of variability in taVNS response, which may include individual differences in nerve anatomy, and differences in baseline brain structure and function. For example, changes in brain structure and function resulting from PD progression might influence taVNS response. There is evidence that non-invasive brain stimulation may lose its effectiveness in modulating monosynaptic targets when white-matter pathways undergo degeneration (48). In contrast with previous non-invasive VNS studies, the results from the current study suggest that bradykinesia and tremor symptoms are the most responsive to stimulation, while gait and posture scores remained relatively unchanged. While improvement on gait and posture items from the UPDRS were not seen in our study, in the only other published taVNS trial for PD, a single 30-min session of stimulation produced a 2.4 point reduction in UPDRS-III and significant improvements in reaction time, gait speed, stride length and swing amplitude (23). In a randomized, sham-controlled transcutaneous cervical VNS trial, a single 2-min session of stimulation produced small improvements in spatiotemporal gait parameters including step length variability and step time (22). Differences in symptom response between this study and others may have been influenced by the overall small numbers of subjects, differences in study inclusion criteria, form of VNS stimulation (auricular vs. cervical) and the parameters used to perform stimulation including intensity, frequency, pulse width, waveform shape, and cycle duration. It is important to consider that while the current taVNS study is the first to use multiple days of stimulation in participants with PD, invasive VNS trials for treatment-resistant depression have demonstrated response rates build over time and can take months to produce meaningful clinical results. Thus, consideration for longer duration trials may be necessary to observe clinically meaningful results (49). A recent pilot study assessed the safety and feasibility of at-home, remotely monitored taVNS for participants to manage long COVID-19 symptoms (50). This remotely monitored approach may offer a solution for providing larger, clinically impactful doses of taVNS and avoid frequent stimulation visits at the study center.

In this study, we did not observe effects on fatigue, sleep impairment, or overall self-reported measures of cognitive functioning; however, a decline in verbal fluency was observed for the taVNS group. Interestingly, this finding shows similarities to the subthalamic nucleus (STN) deep brain stimulation (DBS) literature, wherein the most consistently reported cognitive effect of stimulation is a decline in verbal fluency. This points to the possibility that PD participants may be particularly susceptible to the interruption of brain networks involved in language and speech production. In other patient populations, such as those with treatment-resistant depression, traditional VNS improved verbal fluency (51). Notably, verbal fluency testing was performed at visit 9 shortly within minutes of completing stimulation. Given that there was no indication of subjective decline in cognition on formal scales (CAARS-S:S or PROMIS Applied Cognition), we suspect that this finding might represent an acute effect of stimulation. However, because we did not reassess at the safety follow-up, the time course for resolution is unknown.

A few limitations should be considered when interpreting result from this clinical trial. In this study, taVNS stimulation parameters (i.e., amplitude, frequency, electrode montage, and duty cycle) were chosen based off a systematic assessment in a small group of healthy controls (27). As a result, it is unknown whether stimulation administration was optimized for the PD population where neurodegeneration may impact the dose and stimulation parameters required to achieve therapeutic effects. Although the duration of stimulation in this taVNS study was significantly longer than previous clinical trials, preclinical studies have utilized longer stimulation to achieve physiological benefits. To account for the influence of disease state on response to stimulation, future studies should consider a systematic approach to identify optimal parameters in PD participants. Target engagement studies using neuroimaging and neurophysiological measures can be used to determine stimulation parameters as well as ear target (left vs. right vs. bilateral) which optimally engage afferent targets (26, 52). For example, iterative testing of these various parameters can be evaluated in the context of their ability to elicit changes in markers of vagal tone (i.e., pupil dilation) or blood oxygen level-dependent response within specific brain regions or networks (53). Furthermore, studies using direct neurophysiological measures of neural activity in the subthalamic nucleus via local field potential recordings have been proposed to provide mechanistic insights (54). Thus, future taVNS clinical trials should consider the use of objective these biomarkers to quantify target engagement.

To our knowledge, this is the first study which has evaluated the feasibility, safety, tolerability, and efficacy of multiday taVNS in PD participants. These results suggest the need for an improved mechanistic understanding of taVNS and optimization of stimulation parameters to effectively engage relevant pathophysiological targets (i.e., LC) for the development of future non-invasive VNS clinical trials for PD.

## 5. Conclusions

taVNS is a feasible, well-tolerated and safe neuromodulation approach for individuals with mild to moderate PD. Ten days of taVNS stimulation does not significantly improve global PD motor symptom severity; however, bradykinesia and tremor may be improved by stimulation in a subset of patients. Verbal fluency may be susceptible to transient worsening and should be closely monitored in future taVNS studies. Future randomized clinical trials of taVNS which aim to improve motor and non-motor symptoms in PD will benefit from the establishment of stimulation parameters which optimally engage neural targets, and an at-home treatment paradigm to improve patient centered treatment delivery.

## Data availability statement

The raw data supporting the conclusions of this article will be made available by the authors, without undue reservation.

## Ethics statement

The studies involving human participants were reviewed and approved by Medical University of South Carolina IRB. The patients/participants provided their written informed consent to participate in this study.

## Author contributions

DL: statistical analysis: execution, review, and critique and manuscript: writing of the first draft. TT: research project: execution, statistical analysis: review and critique, and manuscript: review and critique. CM and LL: research project: execution and manuscript: review and critique. HB and BB: research project: conception and manuscript: review and critique. LH: research project: organization and execution and manuscript: review and critique. JE and AP: statistical analysis: design and execution. VH: research project: conception and organization and manuscript: review and critique. All authors contributed to the article and approved the submitted version.

## Supplementary material

Supplementary Figure 1 Individual sensory thresholds used for dosing across participants.
