# Supplementary figures and images for: Multi-session transcutaneous auricular vagus nerve stimulation for Parkinson's disease: evaluating feasibility, safety, and preliminary efficacy
Source: Front Neurol. 2023 Jul 18;14:1210103. doi: 10.3389/fneur.2023.1210103 (PMC10406445; doi:10.3389/fneur.2023.1210103)

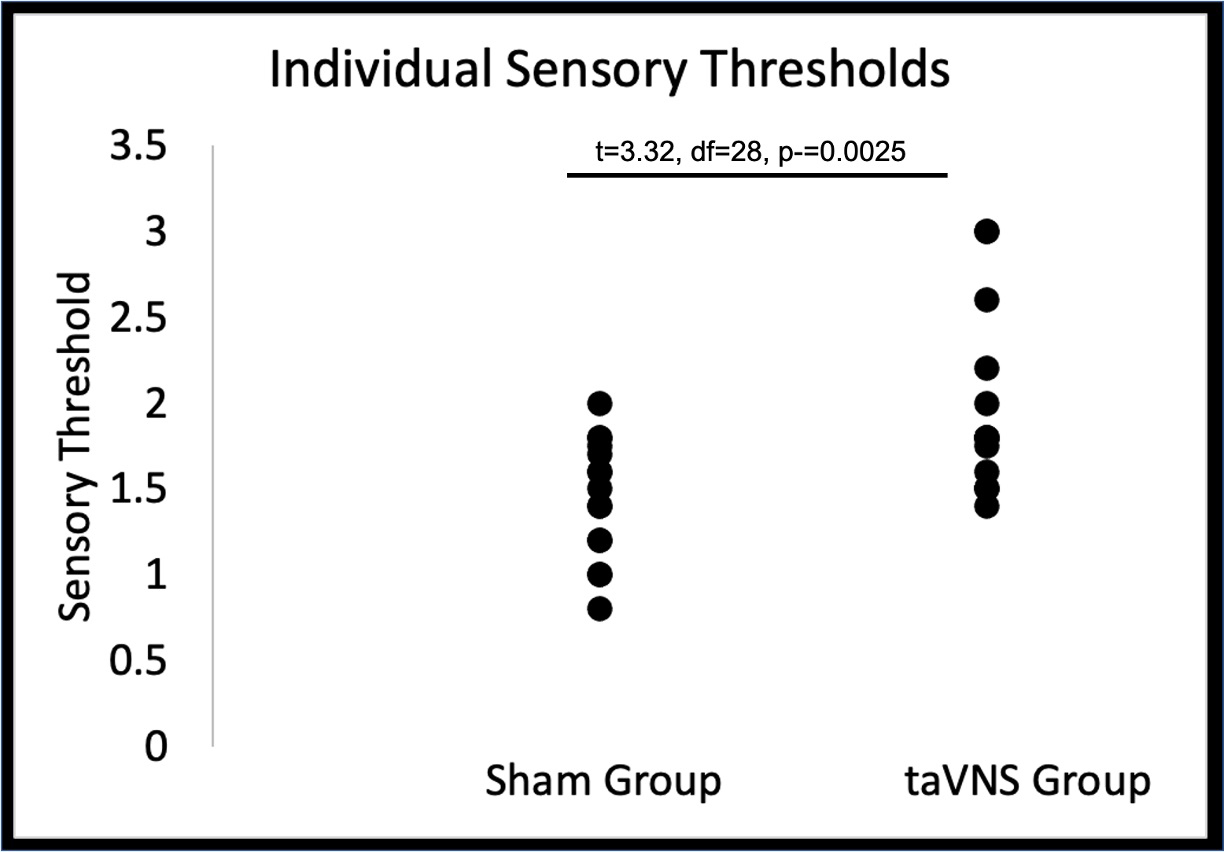

Supplement: Supplementary Figure 1 — Individual sensory thresholds used for dosing across participants. [file Image_1.JPEG]
